# A Framework for an Intelligent and Personalized Fire Evacuation Management System

**DOI:** 10.3390/s19143128

**Published:** 2019-07-16

**Authors:** Jinyue Zhang, Jianing Guo, Haiming Xiong, Xiangchi Liu, Daxin Zhang

**Affiliations:** 1 Tianjin University-Trimble Joint Laboratory for BIM, Department of Construction Management, Tianjin University, 92 Weijin Road, Tianjin 300072, China; 2 China Railway Construction Group Co., Ltd., 20 Shijingshan Road, Beijing 100040, China; 3 Guangxi Hualan Engineering Management Co., Ltd., 23-39 Huadong Road, Nanning 530011, Guangxi, China; 4 Tianjin University Research Institute of Architectural Design and Urban Planning, Tianjin University, 192 Anshanxi Road, Tianjin 300073, China

**Keywords:** fire evacuation management, building information modeling, real-time location system, smartphone, personalized routing

## Abstract

Many research studies have focused on fire evacuation planning. However, because of the uncertainties in fire development, there is no perfect solution. This research proposes a fire evacuation management framework which takes advantage of an information-rich building information modeling (BIM) model and a Bluetooth low energy (BLE)-based indoor real-time location system (RTLS) to dynamically push personalized evacuation route recommendations and turn-by-turn guidance to the smartphone of a building occupant. The risk score (RS) for each possible route is evaluated as a weighted summation of risk level index values of all risk factors for all segments along the route, and the route with the lowest RS is recommended to the evacuee. The system will automatically re-evaluate all routes every 2 s based on the most updated information, and the evacuee will be notified if a new and safer route becomes available. A case study with two testing scenarios was conducted for a commercial office building in Tianjin, China, in order to verify this framework.

## 1. Introduction

Urban fires can result in serious injury or death to human occupants. A total of 1.319 million fires were reported in 2017 in the USA, resulting in 3400 fatalities, 14,670 injuries, and a financial loss of 23 billion US dollars [1]; in China, the 237,000 fires reported in 2018 resulted in 1407 fatalities, 798 injuries, and a loss of 3.67 billion Chinese Yuan [2]. When facing a fire, the most crucial aspect for building safety is the possibility of achieving a safe evacuation of all occupants of the building [3]. In the early stages of a fire event, the building occupants must typically rely on themselves, with a precondition that fire safety facilities in the building (such as fire extinguishers) can provide adequate fire response performance [4]. In practice, measures currently required by design codes do not always work as expected [5], due to inadequate facility maintenance or a lack of operational skills on the part of the occupants. Human behavior during this initial phase becomes a very important factor in the survival of the occupants [6,7], as evacuation behavior reflects how people will behave during an escape and determines the actions they will take, based on their perceptions of the situation [3].

The study of building evacuation stretches back to the early 20th century [8], a time when researchers believed that the movement of occupants in a built environment (such as corridors and staircases) was mainly affected by technical aspects. The relationship between travel speed and occupant density in a physical space with certain dimensions was extensively studied [9,10,11], which laid the foundation for many building design regulations in the field of fire safety, such as the minimum width of evacuation staircases. At the end of the 20th century, human psychology and facility management theories started to be recognized by fire safety researchers and integrated with architectural and engineering aspects [12,13]. Occupancy reflects a relationship between people and their environments; as such, a safe escape not only depends on the personal characteristics of the occupants and the technical parameters of a building, but also how occupants perceive and understand their environments: for example, their familiarity with the floor plan, awareness of fire safety facilities, awareness of fire and smoke growth trends, and so on.

Building information modeling (BIM) is a “modelling technology and associated set of processes to produce, communicate, and analyze building models” used in the architecture, engineering, construction, and operation (AECO) industries [14]. Numerous research studies have investigated the use of BIM to improve project delivery performance, and some BIM-based applications are mature and have been widely used in practice, such as clash detection in design and construction [15], four-dimensional (4D) schedule visualization and optimization [16], and quantity take-offs [17]. Recent relevant research indicates that the lack of real-time, two-way information updates between building occupants and external control centers has caused delays in evacuation, resulting in a high number of deaths or injuries to people in buildings [11,18]. As such, researchers across the world have begun to work on BIM-based fire safety management in the past decade [19], mainly focusing on providing three-dimensional (3D) geometric data to support real-time information retrieval and evacuation simulation. Li et al. [20] used the BIM model to improve the accuracy of room-level localization of trapped occupants, while Ruppel and Schatz [21] developed an interactive game in a BIM-based virtual environment to investigate human behavior during the evacuation process under various emergency scenarios.

Many BIM-based fire management systems have been combined with other information and communication technologies (ICT) to great advantage, for example, sensing systems. In the field of fire safety, temperature sensors and carbon monoxide (CO) sensors are largely used for real-time fire detection [22,23] or evacuation planning [24]. Real-time indoor localization technology is another promising technology that has increasingly been adopted in the AECO industries [25,26,27], including fire safety management. Li et al. [28] developed an environment-aware, sequence-based location (EASBL) to provide building occupants, especially first responders, with timely access to location information. Ruppel et al. [29] designed a system to help emergency rescuers to find the shortest way to a location in a complex building; the developed system combines ultra-wide band (UWB), wireless local area network (WLAN), and radio frequency identification (RFID) to establish a real-time location system. The development and application of mobile Internet provides smartphones with a number of potential uses, including emergency evacuation and management. Inoue et al. [30] proposed a solution for indoor emergency evacuation using various sensors and smartphones. However, this solution does not dynamically determine a new route if an unexpected incident occurs along the first escape route.

There are always uncertainties in the development of a fire, and a better fire evacuation management system should be able to handle the changing conditions in a building environment during a fire. The research presented in this study investigates the most appropriate technology for an indoor real-time location system (TRLS) and the best approach to use for implementation of a BIM model in the cloud to support a mobile application that enables real-time fire evacuation route recommendation, along with turn-by-turn navigation guidance. The risk score (RS) for each possible route is evaluated as a weighted summation of risk level index values of all risk factors for all segments along the route, and the route with the lowest RS is recommended to the evacuee. The system will automatically re-evaluate all routes every few seconds based on the most updated information, either in real-time from sensors or from a database of simulated data. The evacuee will be notified of a new and safer route if one becomes available.

## 2. Materials and Methods

### 2.1. Methodology and System Framework

The purpose of this research was to investigate a better fire evacuation route management system that can handle changing environmental conditions as a fire develops, and to provide a building occupant with turn-by-turn navigation guidance through his/her smartphone. Such a system is comparable to an automobile navigation system that can recommend a new route to the driver of a car if congestion is detected along the current route.

In order to achieve the above-mentioned car navigation system, the system needs a map, a global positioning system (GPS) to determine the driver’s current position, real-time traffic information, a route calculation engine to evaluate possible routes, and a display device that provides a map to the driver, as well as turn-by-turn navigation guidance that directs the driver to follow the recommended route. Similarly, in this research, a cloud-based BIM model serves as the map, an indoor localization system serves as the GPS, real-time data from building sensors or simulated data is used to update the system with the most current condition on the route, an evacuation route calculation and evaluation algorithm is used to recommend an optimized route, and a smartphone serves as the display and notification device.

**Figure 1:**
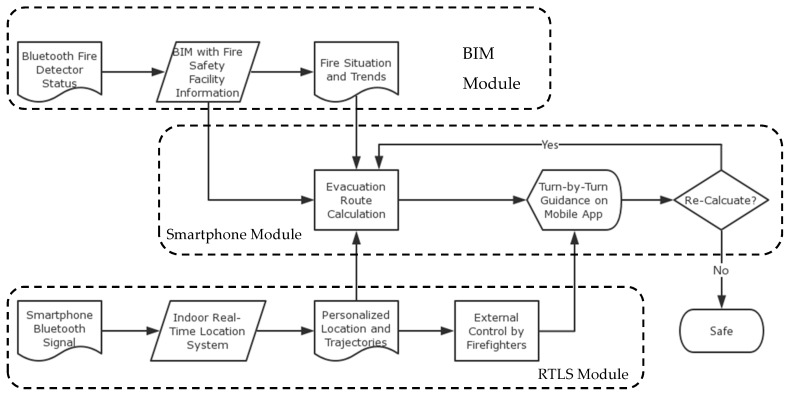
The framework of the proposed intelligent and real-time fire evacuation management system.

According to this methodology, Figure 1 demonstrates the framework for the proposed intelligent and personalized fire evacuation management system. The BIM module is used to understand the fire situation and its spreading trends by combining information obtained from a set of Bluetooth enabled sensors with information about the building’s fire safety facilities that is stored in a BIM model. An real-time location system (RTLS) module is used to determine the indoor locations of building occupants. The locations and trajectories of movement for the occupants are sent to a cloud-based evacuation route calculation processer as well as an external control center staffed by firefighters. Finally, the smartphone module serves as a direct interface between the building occupants and the fire evacuation management system. Based on the real-time location of an occupant, the fire’s current situation and the trends for fire spreading, as well as the information regarding fire safety facilities (such as locations of emergency exits) that is embedded in the BIM model, a safe evacuation route is calculated. This route may not necessarily be the shortest route, but it is the safest one, and personalized turn-by-turn guidance is provided to mobile users. Meanwhile, a user (e.g., a firefighter) at the external control center has the power to overwrite the route as necessary, for example, in the case that the system generates a wrong route. This calculated route may need to be updated in case the fire situation changes and the current route is no longer safe. The system ends when the building occupant is safely evacuated.

### 2.2. BIM Module

As described in Figure 1, the status for the building’s fire detectors is sent to a BIM model in which the locations of fire detectors are modeled. Using the sequence of triggering of the fire detectors, the spreading trends for fire and smoke are predicted. All other fire-safety-related information and data (such as the location of fire exits), which have been documented in the BIM model, are sent to the smartphone module to support the calculation of possible fire evacuation routes.

An information-rich BIM model lies at the core of this system. BIM models are not typically developed for fire safety management, and they are not able to be directly used for fire evacuation management. Because of the intrinsic nature of their 3D visualization feature and their capability for automated error correction empowered by increasingly improved rules defined in BIM authoring tools, BIM models were first developed by designers to coordinate information flow in the design process and ensure a better quality of design deliverables that averts low-level design mistakes or conflicts [14]. Later, contractors developed BIM models that were construction-specific, either by editing existing BIM models or creating new ones from scratch [14]. The BIM models created by contractors are used for trade collaboration, schedule simulation, cost management, construction method simulation, and so on. Not all buildings are BIM ready. For older buildings, it is very likely that a BIM model will need to be developed from the scratch. With the help of modern BIM authoring software, this would not be difficult, because design and construction details for the building are not required—only the 3D geometry data for the building is needed, along with fire-related information such as the locations of fireproof doors.

BIM models for design and construction are usually heavyweight models that require high performance workstations to manipulate. These BIM models are not suitable for the fire safety management application proposed in this paper for two reasons: (1) heavyweight models cannot run on smartphones because they include too much redundant design/construction information that is undesirable for fire safety management applications, and (2) some key information needed for fire safety management may be missing from the design/construction models.

In order to address these two issues, the first need is to find a cloud-based approach to handle lightweight BIM models and support cross-platform applications. At the same time, there is a need to sort the information required for fire safety management so that it can be included in the BIM model.

#### 2.2.1. Cloud-Based Visualization of BIM Models

The online visualization of BIM models that integrate fire safety-related information is different from that for design and construction BIM models on local workstations in two respects [31]. First, an online BIM model needs to be significantly lighter in weight compared to a local BIM model, which could be more than a few hundred megabytes in size. Until 5G mobile Internet can be realized, lightweight BIM data is necessary to improve the user experience for the visualization system. Second, an online BIM model needs to support a number of different operating systems (OSs). Building occupants may use smartphones that run on Apple iOS or Google Android, and the external fire emergency control center could be using either Microsoft Windows or an Apple macOS. It is vital for these different platforms to collaborate and exchange information in a seamless way.

Many 3D engines, including *Unity* by Unity Technologies, support cross-platform development. However, most of those technologies require adjustments in order to meet the requirements of different OS environments; thus, visualization development is typically a customized project rather than a generalized platform. For example, the BIM model of a certain building is imported into a *Unity* project, and then the desired functions are developed. In contrast, Web Graphic Library (WebGL) is a cross-platform and royalty-free application programming interface (API) used to create 3D graphics in a web browser [32], and some researchers have employed such a system. For example, Xu et al. [33] proposed a method to create 3D web visualization for BIM models by combining the Industry Foundation Classes (IFC) data model and WebGL technology. Similarly, Chen et al. [34] proposed a cloud-based system framework to view, store, and analyze massive BIM models, mainly for facility management purposes. There are also several commercial WebGL-based BIM cloud platforms available in the market, including *Forge Viewer* by Autodesk and *BIMFace* by Glodon.

**Figure 2:**
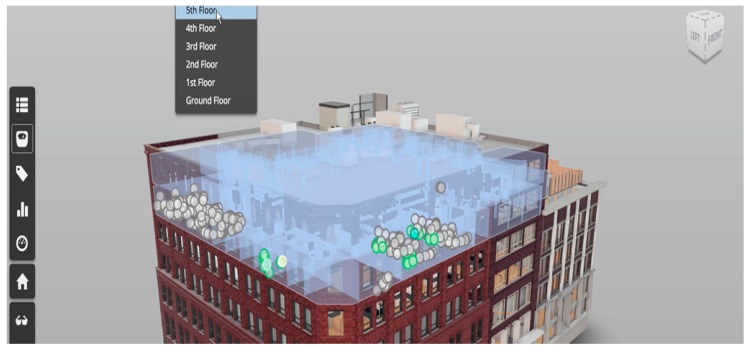
User interface for *Forge Viewer*.

This framework employs a mature platform, *Forge Viewer*, for cloud-based BIM visualization. Before it was officially given the name *Forge Viewer*, Autodesk marketed this product as *Large Model Viewer* because of its capability to handle 3D models larger than those that could be handled by Autodesk’s *Design Review*. The viewer takes a simple vector format (SVF) file and converts it to WebGL format for the browser to display; this enables any browser user with a device that runs any OS to view the information without having to download and install an additional plug-in. Figure 2 is an example of the user interface of *Forge Viewer*. Users can perform many operations in the viewer, such as display the entity’s information, pan/zoom/rotate the view, hide/unhide entities, roam from a first-person perspective, show explosive views, and even cut sections. Currently, *Forge Viewer* supports many popular BIM model formats such as Autodesk *Revit* files, Bentley *MicroStation* DGN files, and many other file formats. Any format not directly supported by *Forge Viewer* can be exported to IFC file format and imported into *Forge Viewer*.

#### 2.2.2. Fire-Safety-Related Information

A lightweight BIM model only ensures smooth operation on a browser; it does not necessarily include all fire-safety-related information in the model. In order to include all the required data in the BIM model, a two-step process is employed: (1) all fire-safety-related information needs to be identified, and (2) all required objects need to be modeled and have all values for their fire-safety-related properties assigned to them.

Based on the 2018 International Fire Code [35], all information categories related to fire safety management were investigated and classified into two groups:

- Required data: Information in this group is definitely required by the proposed fire evacuation management system. This information includes, for example, the location of smoke detectors.
- Recommended data: Information in this group is not directly related to the building evacuation but is recommended for inclusion in the BIM models, as it provides additional reference information that can support better rescue. This information would include, for example, the fire resistance ratings of the major structural components of the building.

The reason that recommended data are differentiated from required data is that some smartphone users may want to keep the size of the building model in their phones to a minimum. Take the fire resistance ratings for major structural components as an example: these ratings can provide firefighters with useful information for selecting a safe route to extinguish a fire, but they are not directly related to the evacuation of the building in the early stages of a fire. A model received for modification and use for this fire evacuation system could be either a design model, a construction model, or a facility management model, and the types of information contained in these models could vary widely. It is necessary to first remove any data that are not related to fire safety management, such as data on the detailed decorations on the walls of the building. Next, all required data and recommended data should be added to the model, but in two separate groups, so as to allow users to select the appropriate data when downloading the model. In total, 56 information categories for required data and 31 information categories for recommended data are identified in a hierarchical structure.

Autodesk *Revit* was selected as the model authoring tool in this study for creating a BIM model for a fire evacuation management system because both *Revit* and *Forge* are products developed by Autodesk, and a *Revit* model is well supported by the *Forge* platform. Some fire-safety-related objects (such as sprinklers) are already pre-defined in *Revit*. Objects that are not pre-defined in *Revit* (such as smoke detectors) might be found in some object libraries such as Autodesk *Seek*; otherwise, they should be created as a new object family that can be reused in multiple BIM projects. In the last step, all required and recommended data are identified. These data include both object types and their properties. For pre-defined objects in *Revit*, properties related to fire safety may or may not be part of the object definition. For example, two key properties included in *Revit* definition of sprinklers are *Pressure Drop* and *Flow*. However, if some properties are not defined in those objects but are required or recommended for use in the fire evacuation management system, for example, *Color Code* and *Water Pressure* for sprinklers, they will need to be added to the object definition through the addition of a custom property set by IFC property set definition. For objects that are not pre-defined in *Revit*, all required/recommended properties must be manually created using the IFC property set definition.

### 2.3. RTLS Module

The RTLS module is used to locate building occupants in real time and, in turn, to support evacuation route calculation in the smartphone module. As shown in Figure 1, Bluetooth signals from the smartphone of a building occupant are captured by Bluetooth signal receivers of an indoor RTLS. Local coordinates (the position with respect to the signal receivers) of the occupant being tracked are calculated by the received signal strength (RSS) method; based on the location of the signal receivers in the building model, the global coordinates (position with respect to the building) of the occupant can be determined. Using these coordinates, the trajectory of the occupant can also be calculated. The trajectories may not be very useful in the real-time evacuation management, but will be very important for post-analysis after a fire accident in order to support further improvements in building design, fire safety facility management, and evacuation planning. The locations of building occupants are sent to the smartphone module for calculation of evacuation routes, but they are also sent to the external control center operated by firefighters to facilitate human intervention in cases where a calculated evacuation route is not the best choice for a safe route.

A reliable indoor RTLS is the key element of the RTLS module. The following section will discuss the possible technologies of an indoor RTLS and the selection of a RTLS for this fire evacuation management system.

#### 2.3.1. Available Indoor RTLS Solutions

RTLS technologies have been investigated for a number of application purposes, including facility management, construction management, sales promotion, and fire safety management [36].

Radio frequency identification (RFID) has been reported to be used in most RTLS solutions because of its cost-effective and flexible approach for identifying individual objects, including people. RFID has an accuracy range of 1 to 3 m and is typically used indoors, whereas the Global Positioning System (GPS) has an accuracy range of 5 to 10 m and is generally used in outdoor environments. For purposes of fire evacuation management, it is not practical to have everyone in a building wearing a RFID tag. It is possible in the case of an office building where the majority of building occupants are regular users of the building. However, in the case of a shopping mall or a hospital, one cannot assume the same scenario.

Ultra-wide band (UWB) technology is very accurate (with an accuracy in the range of 0.1 to 0.3 m), has a short response time, and can be applied for both indoor and outdoor sites. However, while its accuracy is unmatched, the cost of implementing a UWB solution can be prohibitively high [3].

In contrast to the RFID and UWB approaches, vision analysis does not require any devices to be attached to the objects being tracked; however, it requires access to extensive labelled training data sets before implementation, and the inefficiency of vision analysis in dark or dusty environments limits its use in a fire evacuation scenario, where fire and smoke can have a serious adverse impact on the accuracy of a vision-based location system.

**Figure 3:**
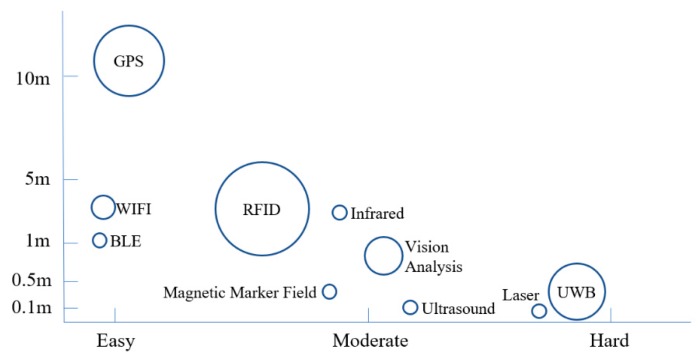
Comparison of major RTLS technologies (where the circle size reflects how widely the technology has been adopted).

Some other indoor positioning technologies—including Bluetooth low energy (BLE), wireless local area networks (WLAN), ultrasound, lasers, radar, infrared, and magnetic marker fields—are also available, and each has its own advantages and limitations. Based on studies of RTLS technologies found in the literature, the authors developed a comparison of major RTLS technologies (shown in Figure 3) in terms of the range of accuracy (as indicated on the vertical axis) and the ease of implementation (both technical and economic feasibility, as indicated on the horizontal axis). The position of each technology and the sizes of the circles shown on this figure are relative, not based on exact values.

#### 2.3.2. BLE-Based RTLS

The RTLS developed in this study employs a BLE-based indoor location system. BLE technology is able to communicate contextual information among multiple connected devices (e.g., BLE sensors, mobile devices, and online computers) with minimal infrastructure in terms of size and cost [37]. The low power required for operation gives the tracking device a very long working life. Because of its small size, a BLE device is suitable for use as a wearable device (and can be incorporated into hardhats or safety vests in the construction industry). Despite its great potential, BLE-based RTLS has not received enough attention from researchers and professionals in many application scenarios.

**Figure 4:**
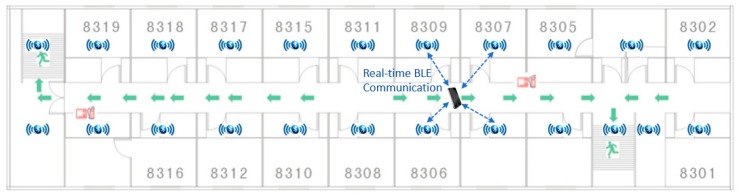
Bluetooth low energy (BLE)-based indoor RTLS.

As shown in Figure 4, Bluetooth sensors are installed in every floor of a building, and their layout depends on the layout for the floor—each Bluetooth sensor typically covers a radius of 5 to 10 m. The coin-sized sensors are about $12 each, their batteries can last for 6–12 months, and they will have minimal impact on the building aesthetics. Regular maintenance of the sensors (for example, checking batteries every 6 months) is required to ensure the batteries are in good condition. A smartphone establishes real-time Bluetooth communication with the surrounding sensors, and the location of the smartphone is determined based on the RSS method.

### 2.4. Smartphone Module

The core function of the smartphone module is to calculate the optimum evacuation route and provide turn-by-turn guidance via a mobile app interface. According to Kobes et al. [4], fire response performance in a building generally depends on three aspects: the characteristics of the fire itself, the characteristics of human occupants, and the characteristics of the building. Building information from the BIM model, information about the fire situation, and the real-time location of the smartphone of a building occupant are the most important inputs needed for the calculation of a possible evacuation route for a given occupant. This research employs the breadth-first search algorithm [38] to generate all possible evacuation routes for evaluation, where the aim is not to find the shortest possible route but the safest one. Route calculation needs to be executed every few seconds based on updated input information until the building occupant is safely outside of the building.

For purpose of evacuation route recommendation in this study, a risk level index (RLI) value from 0 to 4, where 0 indicates the least risk (almost no risk) and 4 indicates the most risk, was assigned to every factor that had an impact on the evaluation of evacuation route. If the risk level was high enough and life-threatening, an RLI value of 500 was assigned to exclude this route from selection. The RLI values were determined by reviewing related literature and conducting a series of interviews with domain experts. Every possible evacuation route for a building occupant was divided into segments, and the risk level for a route was the summation of RLI values of all segments on the route, which were the weighted summations of RLI values of all factors for each segment. The route with the lowest risk level was selected as the evacuation route to be recommended to the building occupant.

#### 2.4.1. Factors Related to Fire

Ambient temperature is one of the key factors for fire evacuation performance, as being exposed to a high temperature level may cause injury and even death [4]. According to Purser [39], a person will have sun shock effects with an ambient temperature value of 60 °C, and is able to resist a temperature value of 82 °C for a duration of 49 min. A temperature value of 148 °C is deemed the limit value for escape, and an RLI value of 500 was assigned to a temperature at that level. Other RLI values for ambient temperature are shown in Table 1. The RLI value used in the calculation was based on the predicted temperature value according to the fire spreading trend estimated in the BIM model.

Carbon monoxide (CO) is a dangerous gas emitted during a fire and, according to Pu and Zlatanova [40], is responsible for more than 50% of the deaths in fires. Atila et al. [41] indicates that there will be no negative effect on human health when the CO density is less than 50 particles per million (ppm). A CO density between 50 ppm and 100 ppm will negatively impact human health, and people will lose consciousness if the CO density reaches between 100 ppm and 3200 ppm and the duration of inhalation is more than 1 h. When the CO density is between 3200 ppm and 12,600 ppm, people will lose consciousness within 30 min. If the CO density exceeds 12,600 ppm, people could die within 3 min. Based on this categorization, RLI values for CO exposure were assigned as shown in Table 1.

Visibility is directly related to the density of smoke caused by a fire and is determined by the growth rate of the fire. The RLI values for various distances of visibility are shown in Table 1.

The value for each fire factor in each segment on the evacuation route that is used to evaluate the total risk level of the route is obtained by environmental sensors or by fire simulation software such as *Fire Dynamics Simulator* (*FDS*), which was used in this research. *FDS* is a computational fluid dynamics model of fire-driven fluid flow, designed by the National Institute of Standard and Technology in the United States. The software executes numerical solutions in the form of Navier–Stokes equations appropriate for thermally driven flow, with an emphasis on smoke and heat transport from fires. The fire-related, information-rich BIM model and the fire parameters entered as the initial inputs are used to simulate fire development and predict the temperature, CO density, and visibility in the building space considered for evacuation [42]. Fire growth rate is a significant parameter in simulating fire development. The National Fire Protection Association defines fire growth rate as the heat released from the fire within one second (expressed in kJ/s^3^), and classifies the fire as ultra-fast, fast, medium, or slow according to its speed [43]. For the starting segment in a selected evacuation route, which is the current location of the building occupant, a real-time sensor value is preferred if available (for example, the CO density from a CO detector). If real-time data are not available, a simulated value will be used (for example, the CO density in a segment where a building occupant will be after 15 s along a selected evacuation route).

It should be noted that a *FDS* simulation usually takes hours to finish. Thus, it is not possible to run a real-time simulation while a fire is occurring, even when using the most powerful cloud-based solution. The approach adopted in this research was to divide the building plan into cells based on the building’s layout. *FDS* simulations were executed for every cell as the fire originated in that cell, and the simulated data were saved in a database to be retrieved on demand. Running *FDS* in the cloud could be much faster than using a single computer because a cloud solution can use more than 100 cores to achieve parallel computing. For this study, Sabalcore’s High Performance Computing Cloud was employed to conduct a total of 26 simulations.

#### 2.4.2. Factors Related to Building Occupants

The total evacuation time for a building occupant depends on the occupant’s speed of movement. Human density, which is defined as the number of people per square meter, is known to have a significant impact on the speed of human movement. People are able to move freely when human density is less than 0.8 person/m^2^. Walking speed will be 1–1.5 m/s when human density is between 0.8 person/m^2^ and 1.8 person/m^2^, and it will drop to 0.5–1 m/s when human density is between 1.8 person/m^2^ and 4 person/m^2^. If human density exceeds 4 person/m^2^, movement stops completely [44]. The risk level index values for human density are shown in Table 2.

Human movement speed in an emergency evacuation also depends on the degree of familiarity with the building’s layout [4]. People who are regular occupants of a building will move faster within a given evacuation route than visitors who are unfamiliar with the building’s layout, because they have no need to stop and look for directions at intersections. In this research, only three values (0, 1, and 2) were assigned to this factor.

People with proper protective equipment (such as gas masks or flame-resistant clothing) will be better protected during evacuation, and thus could endure a more severe environment. In this research, a value of 0 was assigned to people with fire/smoke protection gear and a value of 1 was assigned to people without any fire/smoke protection gear.

#### 2.4.3. Factors Related to Building

The length of an evacuation route from any location in a building to an emergency/safety exit is usually the first consideration when escaping a fire, and this is also the principle for escape planning—for example, indicating an escape route on the door of each hotel room. However, as there are always uncertainties during the development of a fire, a pre-defined escape route could be the shortest available route—but not the safest one—if a fire is located along the escape route or is rapidly approaching it. For this reason, the level of risk was deemed more important than the route length in the evaluation of an evacuation route in this research study. However, the route length remains a factor in selecting the optimum evacuation route. Most researchers consider the entire length of the route as a single factor [45,46,47], although some researchers investigating real-time route optimization have considered changes in conditions along a given route. For example, Han et al. [24] introduced a model to integrate real-time data from temperature sensors and CO sensors in route planning for high-rise buildings. In this research, a route was divided into segments between turning points, such as the horizontal turning points at intersections of hallways or the vertical turning points at staircases. If a segment of a route was longer than 5 m, then it was divided into multiple segments of 5 m or less. The reason for dividing a route is that on a long route, the risk level at different segments of a route could be different, and the risk level at a given segment may change over time; as such, it is necessary to calculate the risk level for each segment separately.

Another factor related to the characteristics of a building is the type of route. People will move faster in a flat corridor than in a staircase, for example, so different types of evacuation routes will have different risk level index values, as shown in Table 3. Other factors such as the availability of emergency lamps or fire sprinklers were also considered in this research, and the index values for these are also provided in Table 3.

#### 2.4.4. Calculation of Total Risk Level

The total risk level for each available evacuation route for a given building occupant is represented by a risk score (RS), which is the summation of RLI values of all factors for all segments of the route. However, the importance of each of abovementioned factors in calculating the total RS is not equal, and there is a need to assign a weight for each factor. A focus group interview was organized, and 14 domain experts were surveyed to determine the weights to use for each risk factor in the calculation of the RS. In the survey, each focus group attendee was asked to rank all nine factors listed in Table 1, Table 2 and Table 3 using a seven point Likert scale. The orders of precedence for all factors are listed in Table 4, and mean values were used as weights in the calculation of the RS.

The RS for each optional evacuation route was calculated by a weighted summation of risk level indexes in all segments on the route:

(1) $$\mathrm{RS}=\sum_{i=1}^{m}\sum_{j=1}^{n}\frac{L_{i}}{5}W_{j}RLI_{j}$$

where: *RS* is the total risk scale of a given evacuation route, *m* is the total number of segments on this route, *n* is the total number of participating factors in the *i*th segment, *L* is the length of the *i*th segment (*L* ≤ 5), *W* is the weight (the mean value in Table 4) of the *j*th participating factor in the *i*th segment, and *RLI* is the risk level index value of the *j*th participating factor in the *i*th segment.

## 3. Results and Discussion

A case study was conducted in a commercial office building in Tianjin, China, to examine the proposed fire evacuation management framework. The case study included (1) BIM model preparation, (2) BLE-based indoor RTLS deployment, and (3) mobile app prototype development.

### 3.1. BIM Model Prepartion

**Figure 5:**
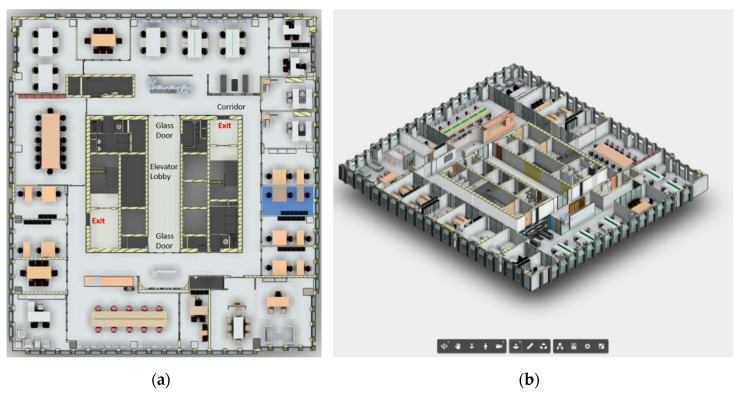
(**a**) Floorplan of the office building, (**b**) building information modeling (BIM) model for the building with navigation tools.

The space used for the case study was the 34th floor of a commercial office building, with the floor plan shown in Figure 5a. The building core included elevators, washrooms, equipment rooms, and two staircases. A circular corridor separated the building core and the office areas, and there were two emergency exits near the two staircases.

A BIM model was created in Autodesk *Revit* by removing irrelevant information from the construction model and adding all required/recommended fire-safety-related information by either expanding the properties using IFC extensions, or by creating new objects and manually adding their properties. This BIM model was then sent to Autodesk *Forge*, and a lightweight 3D model was obtained, as shown in Figure 5b.

A cloud-based server was set up to run *FDS*. The BIM model was exported to an IFC data model; next, based on the method proposed by Dimyadi et al. [48], the IFC data model was used to share building geometry and other information with *FDS*. The statuses of sensors sent to the cloud server were used to simulate fire and smoke development in *FDS*. Sensors installed for this case study included Bluetooth-enabled temperature sensors, smoke detectors, flame detectors, and CO detectors. These sensors were installed in each room (one each per room) and in all corridors (one of each sensor installed every 5 m).

### 3.2. RTLS Deployment

**Figure 6:**
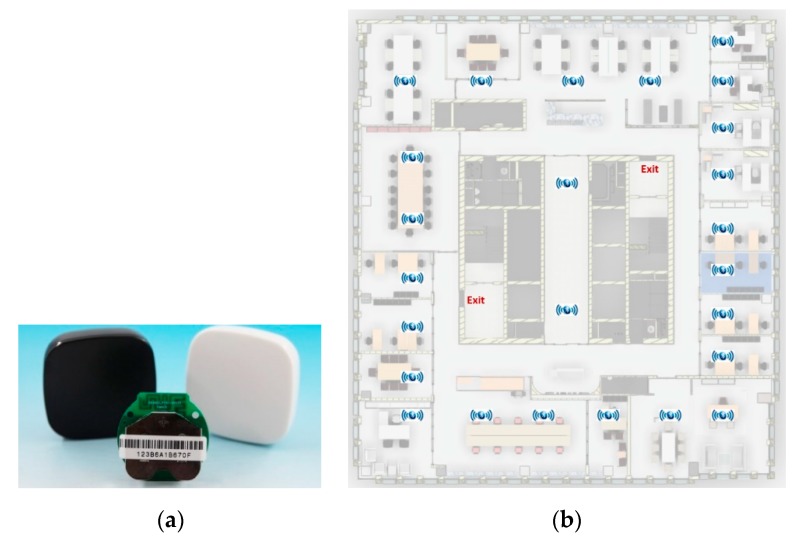
(**a**) Bluetooth sensor employed in this study, (**b**) layout of 26 Bluetooth sensors.

Due to the limited budget, this case study only covered the 34th floor and not the entire building. A Bluetooth sensor, as shown in Figure 6a, can cover a circular area with a radius of 5 to 10 m. A total of 26 Bluetooth sensors were installed, and the layout of sensors is shown in Figure 6b. Bluetooth communication data between the smartphone of a building occupant and the Bluetooth sensors were sent to the cloud server to calculate the location of the building occupant. In terms of the accuracy of indoor localization, a BLE-based system normally has an accuracy range of 1–5 m, depending on the number of sensors and their layout, the number of obstructions and their materials, the power of sensors, etc. After the deployment of this testing system, a calibration process was conducted by comparing the calculated coordinates to the actual coordinates in the building. Thanks to the reinforced concrete frame structure of the building, the partition walls did not present much of an obstruction. As such, this system produced an accuracy in the range of 1.5–1.8 m.

### 3.3. Mobile App Development

In this study, a mobile app prototype was developed on the Android platform. Some functions that are not directly related to validation of the proposed fire evacuation management framework were not developed (for example, account management and BIM model management). It is supposed that a building occupant has already installed this app on his/her smartphone and has set up an account. Working with the GPS function of the smartphone, the app should be able to detect when a user has entered a building with a BIM model that has never been downloaded into the app. The app then pushes a notification to the app user to prompt him/her to download the BIM model for the building from the server and define the values of two factors: “Building Familiarity” and “Fire/Smoke Protection.” For example, a person checking in at a hotel may assign “Not Familiar” to “Building Familiarity” and “Yes” to “Fire/Smoke Protection” after confirming with the hotel’s front desk clerk that smoke masks are available in every room. In this case study, the BIM model has been downloaded, and values of those two factors have been defined.

### 3.4. Test Scenario 1

**Figure 7:**
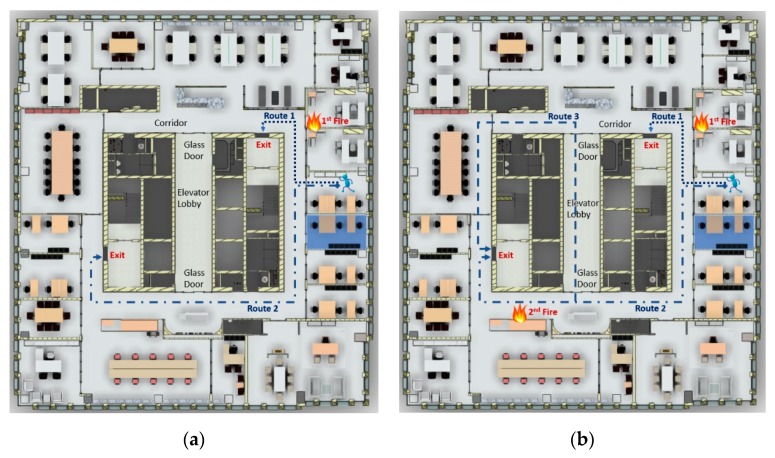
Evacuation route selection in (**a**) Scenario 1 and (**b**) Scenario 2.

**Figure 8:**
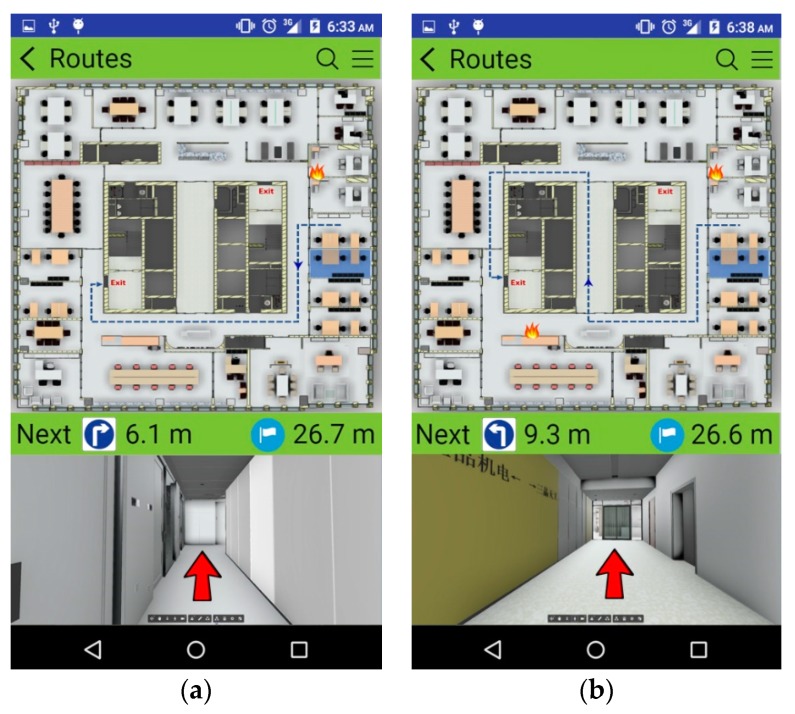
Smartphone display in (**a**) Scenario 1 and (**b**) Scenario 2.

Figure 7a shows the first test scenario, including the location of the building occupant, the location of origin for the fire, and two system-generated evacuation routes. Route 1 is shorter than Route 2, but the origin of the fire is located near a point on Route 1; thus, the building occupant should select Route 2 if he/she completely understands the situation, since the longer route is much safer. The values of *RLI* for risk factors should be determined by either real-time factor values from sensors (at the current time) or predicted (simulated) factor values (at a later time). The speed of movement for the occupant is determined by the real-time human density obtained by the RTLS, which is then used to determine the time at which a building occupant will arrive at a certain segment. Table 5 and Table 6 show the calculation of RS for each route by segment for Route 1 and Route 2, respectively. From the RS values for each route, it can be noticed that Route 2 is a safer choice than Route 1, although taking Route 2 will require 15 s for an occupant to evacuate the 34th floor, compared to 7 s for Route 1. Figure 8a shows the real-time, turn-by-turn indoor navigation as it appeared on the smartphone. The upper half of the screen shows an overview of the evacuation route recommended to the occupant, with a moving arrow indicating the current location of the evacuee. The lower half of the screen shows the 3D space in a first-person perspective powered by Autodesk *Forge,* with a large directional arrow to help guide the evacuee along the selected route. Along with the visual guidance, voice guidance is also provided, which is very important in a case where smoke creates a condition of low visibility.

### 3.5. Test Scenario 2

The proposed system will run the RS calculation every two seconds based on the most updated data to check if the current route is still the optimum escape route. In the first test scenario, no new fire developed during the evacuation. Test Scenario 2 involves the creation of a secondary fire along Route 2 in order to test the recalculation of the evacuation route. The location of the secondary fire, which is shown in Figure 7b, is near Route 2 and originated 5 s after the first fire. Route 3 (as shown in Figure 7b) will not be considered in Test Scenario 1, as it will be much longer than Route 2, it passes by two glass doors at both ends of the elevator lobby, and it heads in the direction of the first fire. However, if the new fire develops, the calculation of RS indicates that Route 3 is safer than Route 2. The calculations for Route 2 and Route 3 are shown in Table 7 and Table 8, respectively. The notification on the smartphone at the 6th s is shown in Figure 8b, advising the occupant to switch from Route 2 to Route 3.

### 3.6. Discussion

The results of the test run indicate that the system was able to determine the safest route in both scenarios, with and without a second fire. The building users did not observe any significant delay in receiving the turn-by-turn navigation guidance. Due to the relatively small building plan and the small number of users, the test run did not induce an unacceptable computing load on the cloud server hosted in Amazon Azure. This study shared a virtual machine with another research group that has a high-performance account, with 16 Intel Xeon E5 2667 processors and 112 GB of memory. In the future, a large-scale test could be conducted to examine the minimum computing requirements for such a system. The authors are confident about the computing time, given recent advances in cloud computing and hardware technology. The data usage and the speed of data transfer also should not be an issue, especially with the rapid adoption of 5G technology.

The proposed fire evacuation management framework largely relies on sensors installed in a building. One could argue that the system will not function in a case where the sensors are damaged in a fire. However, this system is designed to help building occupants at the beginning stages of a fire; as such, it assumed that not all sensors will be damaged while the fire is still developing. In addition, the simulated data could be used in cases where data from a given sensor are lost. A possible future study could focus on the development of an algorithm to generate data for lost signals to increase the robustness of the system. In addition, the loss of power to the building from the electrical grid is not a factor, since all Bluetooth sensors, temperature sensors, and CO sensors are battery-operated. Moreover, a mobile phone will always be able to receive a push notification from the cloud sever so long as the program is initiated when a fire starts.

This research simplifies the assumption regarding the personal physical characteristics of building occupants by assuming that all occupants are healthy people and that there is no difference in speed of movement between people of different genders and ages. In reality, men are typically able to move faster than women, and elderly people will generally be slower than young people [49]. Some building occupants may also have health issues such as joint–muscle disease or respiratory disease. The worst-case situation is an occupant who is physically disabled and moves by using a wheelchair. Future research should take physical characteristics of building occupants into account and adjust the model for the RS calculation accordingly.

In this proposed fire evacuation management framework, an extremely high value of 500 is assigned to any factor with a risk level high enough to jeopardize human life—for example, when the ambient temperature exceeds 148 °C or the CO density is greater than 12,600 ppm in a given segment. This means that if a segment in a given route has life-threatening conditions, it should be eliminated from consideration. However, if the fire situation is extremely serious and there is no alternate route for a building occupant to be evacuated safely, the system should check the situation for all building spaces and find a place which is the least risky based on the *FDS* simulation, and is a location where the occupant can be easily rescued based on information in the BIM model (for example, a room with a window). In this case, the system could suggest a route to a relatively safe location where the building occupant could shelter while awaiting rescue.

Due to the limited budget in the project, in this case study, only a few active building occupants were tested in both testing scenarios. This proposed system is a personalized evacuation system, and thus it is assumed that multiple users will act independently but not produce correlations. As such, the human density value was always less than 0.8 people per square meter. This caused the values of RLI for F5 in all segments in Table 5, Table 6, Table 7 and Table 8 to have a value of 0. As human density is known to play a significant role in determining the speed of movement during an evacuation, future research should involve testing the developed system using different levels of human density in order to calibrate the parameters in Table 2. The authors are planning to deploy the system in a larger scale school building early next year at Tianjin University, so there will be a better chance to test the system under additional scenarios.

## 4. Conclusions

Designing a reliable system to safely evacuate building occupants as quickly as possible is a significant issue in facility design and operation for complex buildings. Compared to residential buildings, commercial and institutional buildings have more complicated structures and complex layouts, mixed-use functions, and a higher number of building occupants who may not be familiar with the building layout and fire safety facilities, and thus need more attention in the development of an effective fire evacuation management system.

This research proposed a fire evacuation management framework that combines the advantages of the information-rich BIM model and a BLE-based indoor RTLS to dynamically push personalized evacuation route recommendations and turn-by-turn guidance to the smartphones of building occupants. A BIM model encapsulates all fire-safety-related information, such as the locations of fire detectors and other sensors, locations of fire extinguishers and sprinklers, fire resistance ratings for building components and goods in the building, along with space geometry and the locations of emergency exits. Based on information from the BIM model, combined with the real-time data obtained from sensors, FDS can be used to simulate the development of fires and predict the temperature, CO density, and visibility at locations throughout the entire building at any point in time. A BLE-based indoor RTLS is very cost effective and easy to deploy. This RTLS communicates with a building occupant’s smartphone, and can locate the occupant in real-time with respect to the space defined in the BIM model. Based on the occupant’s location and building geometry, several possible evacuation routes are generated. The RS for each route is calculated as a weighted summation of the risk level index values for all risk factors in all segments along the route. The route with the lowest RS is recommended to the occupant, and turn-by-turn guidance is displayed on the smartphone of the evacuee along with voice guidance.

A case study was conducted in a commercial office building in Tianjin, China, to verify the proposed fire evacuation management framework. In the first test scenario, only one fire was considered, and a longer but safer route was recommended to the building occupant, because the shorter route would require the evacuee to move towards the fire. In the second test scenario, a secondary fire developed at a location near the chosen route at 5 s after the building occupant began to evacuate along that route. As the system has the ability to re-evaluate all routes every 2 s, a new route was recommended to the evacuee as soon as a safer route was available.

There are two contributions of the proposed fire evacuation management framework. First, it provides an integrated solution that uses the most appropriate technologies in cloud-based BIM, indoor localization systems, and mobile computing. Second, this research proposes the concept of a risk score (RS) for each possible route, which is a weighted summation of the risk level index values of all risk factors for all segments along the route. This framework can be further enhanced by incorporating the physical characteristics of building occupants into the RS calculation, and by defining a policy to handle a situation where no safe evacuation route is available.

There are several potential improvements for the proposed framework. The most significant part is to develop an algorithm that is able to generate data for lost signals to increase the robustness of the system, probably using a machine learning method that analyzes the pattern of data from each sensor. Second, the personal physical characteristics of building occupants should be integrated into the framework to produce a more accurate recommendation based on personal movement capability. Some other potential improvements could be the recommendation of a relatively safe area in the building in which to shelter in a case where there is no safe exit route available, a better indoor localization system, or a feature that allows interaction between building occupants and external rescue workers.

## Figures and Tables

**Table 1:** Factors related to fire and their risk level index (RLI) values.

Factor Name	Risk Level Index Value					
	Index = 0	Index = 1	Index = 2	Index = 3	Index = 4	Index = 500
Temperature (°C)	0–40	41–60	61–80	81–100	100–147	148+
CO density (ppm)	0–50	50–100	100–1000	1000–3200	3200–12,600	12,600+
Visibility (meters)	30+	20–30	10–20	5–10	0–5	

**Table 2:** Factors related to building occupants and their index values.

Factor Name	Risk Level Index Value				
	Index = 0	Index = 1	Index = 2	Index = 3	Index = 4
Human density (person/m^2^)	0–0.8	0.8–1.8	1.8–2.8	2.8–4	4+
Building familiarity	Very Familiar	Familiar	Not Familiar		
Fire/smoke protection	Yes	No			

**Table 3:** Factors related to building and their index values.

Factor Name	Risk Level Index Value				
	Index = 0	Index = 1	Index = 2	Index = 3	Index = 4
Evacuation route type	Room	Corridor	Ramp	Stair	Elevator
Emergency lamp	Yes	No			
Fire sprinkler	Yes	No			

**Table 4:** Mean and standard deviation of all risk factors.

Order	Factor	Mean	Standard Deviation
F1	Temperature	6.34	2.07
F2	Visibility	6.11	2.81
F3	Fire/smoke protection	5.94	2.15
F4	CO density	5.85	2.33
F5	Human density	3.77	2.69
F6	Fire sprinkler	3.36	3.32
F7	Building familiarity	3.28	2.96
F8	Evacuation route type	1.92	2.68
F9	Emergency lamp	1.16	3.22

**Table 5:** RS calculation for Route 1.

S. No	Time	L (m)	F1	F2	F3	F4	F5	F6	F7	F8	F9	W. Sum
1	0:00	4.2	0	0	1	0	0	0	0	0	1	5.96
2	0:02	5	1	1	1	1	0	0	0	1	0	26.16
3	0:04	1.2	3	2	1	2	0	0	0	1	0	12.19
4	0:05	3.8	4	4	1	3	0	0	0	1	0	57.16
TT =	0:07										RS =	101.48

S. No = segment number, Time = time of arrival, L = segment length, F1–F9 are factors listed in Table 4, W. Sum = weighted sum, TT = total time, and RS = Risk Scale.

**Table 6:** RS calculation for Route 2 (without a second fire).

S. No	Time	L (m)	F1	F2	F3	F4	F5	F6	F7	F8	F9	W. Sum
1	0:00	4.2	0	0	1	0	0	0	0	0	1	5.96
2	0:02	5	1	1	1	0	0	0	0	1	0	20.31
3	0:04	5	0	0	1	0	0	0	0	1	0	7.86
4	0:06	0.9	0	0	1	0	0	0	0	1	0	1.41
5	0:06	5	0	0	1	0	0	0	0	1	0	7.86
6	0:08	5	0	0	1	0	0	0	0	1	0	7.86
7	0:10	5	0	0	1	0	0	0	0	1	0	7.86
8	0:12	1.3	0	0	1	0	0	0	0	1	0	2.04
9	0:13	4.3	0	0	1	0	0	0	0	1	0	6.76
TT =	0:15										RS =	67.93

S. No = segment number, Time = time of arrival, L = segment length, F1–F9 are factors listed in Table 4, W. Sum = weighted sum, TT = total time, and RS = Risk Scale.

**Table 7:** RS calculation for Route 2 (including the second fire).

S. No	Time	L (m)	F1	F2	F3	F4	F5	F6	F7	F8	F9	W. Sum
1	0:00	4.2	0	0	1	0	0	0	0	0	1	5.96
2	0:02	5	1	1	1	0	0	0	0	1	0	20.31
3	0:04	5	0	0	1	0	0	0	0	1	0	7.86
4	0:06	0.9	0	0	1	0	0	0	0	1	0	1.41
5	0:06	5	1	0	1	0	0	0	0	1	0	14.20
6	0:08	5	2	1	1	1	0	0	0	1	0	32.50
7	0:10	5	3	2	1	2	0	0	0	1	0	50.80
8	0:12	1.3	3	2	1	2	0	0	0	1	0	13.21
9	0:13	4.3	2	1	1	1	0	0	0	1	0	27.95
TT =	0:15										RS =	174.21

S. No = segment number, Time = time of arrival, L = segment length, F1–F9 are factors listed in Table 4, W. Sum = weighted sum, TT = total time, and RS = Risk Scale.

**Table 8:** RS calculation for Route 3 (in response to the second fire).

S. No	Time	L (m)	F1	F2	F3	F4	F5	F6	F7	F8	F9	W. Sum
1	0:00	4.2	0	0	1	0	0	0	0	0	1	5.96
2	0:02	5	1	1	1	0	0	0	0	1	0	20.31
3	0:04	5	0	0	1	0	0	0	0	1	0	7.86
4	0:06	0.9	0	0	1	0	0	0	0	1	0	1.41
5	0:06	5	1	0	1	0	0	0	0	1	0	14.20
6	0:08	2.7	2	1	1	1	0	0	0	1	0	17.55
7	0:10	5	1	0	1	0	0	0	0	1	0	14.20
8	0:12	5	0	0	1	0	0	0	0	1	0	7.86
9	0:14	4.1	1	0	1	0	0	0	0	1	0	11.64
10	0:15	5	1	0	1	0	0	0	0	1	0	14.20
11	0:17	3.8	0	0	1	0	0	0	0	1	0	5.97
12	0:18	5	1	0	1	0	0	0	0	1	0	14.20
13	0:20	3.5	2	1	1	1	0	0	0	1	0	22.75
TT =	0:21										RS =	158.13

S. No = segment number, Time = time of arrival, L = segment length, F1–F9 are factors listed in Table 4, W. Sum = weighted sum, TT = total time, and RS = Risk Scale.

## References

[1] US Fire Administration U.S. Fire Statistics. https://www.usfa.fema.gov/data/statistics/.

[2] Ministry of Emergency Management Fire Administration Department National Fire and Police Situation in 2018. http://www.119.gov.cn/xiaofang/hztj/36306.htm.

[3] Deak G., Curran K., Condell J. (2012). A survey of active and passive indoor localisation systems. Comput. Commun..

[4] Kobes M., Helsloot I., De Vries B., Post J.G. (2010). Building safety and human behaviour in fire: A literature review. Fire Saf. J..

[5] Rubadiri L., Ndumu D.T., Roberts J.P. (1997). Predicting the evacuation capability of mobility-impaired occupants. Fire Technol..

[6] Sierra F.J.M., Rubio-Romero J.C., Gámez M.C.R. (2012). Status of facilities for fire safety in hotels. Saf. Sci..

[7] Purser D.A., Bensilum M. (2001). Quantification of behaviour for engineering design standards and escape time calculations. Saf. Sci..

[8] Pires T.T. (2005). An approach for modeling human cognitive behavior in evacuation models. Fire Saf. J..

[9] O’Connor D.J. (2005). Integrating human behaviour factors into design. J. Fire Prot. Eng..

[10] Frantzich H. (1994). A Model for Performance-Based Design of Escape Routes.

[11] Fahy R.F., Proulxm G. Toward creating a database on delay times to start evacuation and walking speeds for use in evacuation modeling. Proceedings of the Second International Symposium on Human Behaviour in Fire.

[12] Society of Fire Protection Engineers (SFPE) (2002). Engineering Guide to Human Behaviour in Fire.

[13] Sime J.D. (2001). An occupant response shelter escape time (ORSET) model. Saf. Sci..

[14] Eastman C., Teicholz P., Sacks R., Liston K. (2011). BIM Handbook: A Guide to Building Information Modeling for Owners, Managers, Designers, Engineers, and Contractors.

[15] Hu Y., Castro-Lacouture D., Eastman C.M. (2019). Holistic clash detection improvement using a component dependent network in BIM projects. Automat. Constr..

[16] Koo B., Fisher M. (2000). Feasibility study of 4D CAD in commercial construction. J. Constr. Eng. Manag..

[17] Monteiro A., Martins J.P. (2013). Survey on modeling guidelines for quantity takeoff-oriented BIM-based design. Automat. Constr..

[18] Gershon R.R.M., Magda L.A., Riley H.E.M., Sherman M.F. (2012). The World Trade Center evacuation study: Factors associated with initiation and length of time for evacuation. Fire Mater..

[19] Wang S.H., Wang W.C., Wang K.C., Shih S.Y. (2015). Applying building information modeling to support fire safety management. Automat. Constr..

[20] Li N., Becerik-Gerberb B., Krishnamacharic B., Soibelmanb L. (2014). A BIM centered indoor localization algorithm to support building fire emergency response operations. Automat. Constr..

[21] Rüppel U., Schatz K. (2011). Designing a BIM-based serious game for fire safety evacuation Simulation. Adv. Eng. Inform..

[22] Kuo H.C., Chang H.K. (2003). A real-time shipboard fire-detection system based on grey-fuzzy algorithms. Fire Saf. J..

[23] Han D., Lee B. (2009). Flame and smoke detection method for early real-time detection of a tunnel fire. Fire Saf. J..

[24] Han Z., Weng W., Zhao Q., Ma X., Liu Q., Huang Q. (2013). Investigation on an integrated evacuation route planning method based on real-time data acquisition for high-rise building fire. IEEE Trans. Intell. Transp. Syst..

[25] Costin A.M., Teizer J. (2015). Fusing passive RFID and BIM for increased accuracy in indoor localization. Vis. Eng..

[26] Park J., Chen J., Cho Y.K. (2017). Self-corrective knowledge-based hybrid tracking system using BIM and multimodal sensors. Adv. Eng. Inform..

[27] Xue F., Chen K., Lu W., Niu Y., Huang G.Q. (2018). Linking radio-frequency identification to Building Information Modeling: Status quo, development trajectory and guidelines for practitioners. Automat. Constr..

[28] Li N., Yang Z., Ghahramani A., Becerik-Gerber B., Soibelman L. (2014). Situational awareness for supporting building fire emergency response: Information needs, information sources and implementation requirements. Fire Saf. J..

[29] Rüppel U., Stubbe K.M., Zwinger U. Indoor navigation integration platform for firefighting purposes. Proceedings of the 2010 International Conference on Indoor Positioning and Indoor Navigation (IPIN 2010).

[30] Inoue Y., Sashima A., Ikeda T., Kurumatani K. Indoor emergency evacuation service on autonomous navigation system using mobile phone. Proceedings of the Universal Communication Second International Symposium on IEEE.

[31] Zhou X., Wang J., Guo M., Gao Z. (2018). Cross-platform online visualization system for open BIM based on WebGL. Multimed. Tools Appl..

[32] Parisi T. (2012). WebGL: Up and Running.

[33] Xu Z., Zhang Y., Xu X. (2016). 3D visualization for building information models based upon IFC and WebGL integration. Multimed. Tools Appl..

[34] Chen H.M., Chang K.C., Lin T.H. (2016). A cloud-based system framework for performing online viewing, storage, and analysis on big data of massive BIMs. Automat. Constr..

[35] International Code Council, Inc. (ICC) International Fire Code. https://www.iccsafe.org/codes-tech-support/codes/2018-i-codes/ifc/.

[36] Li H., Chan G., Wong J.K.W., Skitmore M. (2016). Real-time locating systems applications in construction. Automat. Constr..

[37] Park J., Kim K., Cho Y.K. (2016). Framework of automated construction-safety monitoring using cloud-enabled BIM and BLE mobile tracking sensors. J. Constr. Eng. Manag..

[38] Russell S.J., Norvig P. (2016). Artificial intelligence: A Modern Approach.

[39] Purser D.A. (2008). Toxicity assessment of combustion products. SFPE Handbook of Fire Protection Engineering.

[40] Pu S., Zlatanova S. (2005). Evacuation route calculation of inner buildings. Geo-Information for Disaster Management.

[41] Atila U., Ortakci Y., Ozacar K., Demiral E., Karas I. (2018). SmartEscape: A Mobile Smart Individual Fire Evacuation System Based on 3D Spatial Model. ISPRS Int. J. Geo-Inf..

[42] McGrattan K., Hostikka S., McDermott R., Floyd J., Weinschenk C., Overholt K. Fire Dynamics Simulator User’s Guide. https://ws680.nist.gov/publication/get_pdf.cfm?pub_id=913619.

[43] National Fire Protection Association (NFPA) (2009). Standard for Smoke Management Systems in Malls, Atria, and Large Spaces.

[44] Milinskii A.I. (1951). The Study of Egress Processes from Public Buildings of Mass Use. Ph.D. Thesis.

[45] García-Ojeda J.C., Bertok B., Friedler F., Fan L.T. (2013). Building-evacuation-route planning via time-expanded process-network synthesis. Fire Saf. J..

[46] Capote J.A., Alvear D., Abreu O., Cuesta A., Alonso V. (2012). A stochastic approach for simulating human behaviour during evacuation process in passenger trains. Fire Technol..

[47] Capote J.A., Alvear D., Abreu O., Cuesta A. (2012). Analysis of evacuation procedures in high-speed trains fires. Fire Saf. J..

[48] Dimyadi J., Spearpoint M., Amor R. (2008). Sharing building information using the IFC data model for FDS fire simulation. Fire Saf. Sci..

[49] Hodgkins J. (1963). Reaction time and speed of movement in males and females of various ages. Res. Q. Am. Assoc. Health Phys. Educ..

